# In at the deep end: the physiological challenges associated with artistic swimming

**DOI:** 10.1007/s10286-024-01070-z

**Published:** 2024-10-04

**Authors:** E. L. Williams, C. J. Mathias, S. Sanatani, M. J. Tipton, V. E. Claydon

**Affiliations:** 1 Department of Biomedical Physiology and Kinesiology, Simon Fraser University, Burnaby, BC Canada; 2 BC Artistic Swimming, New Westminster, British Columbia Canada; 3 Autonomic and Neurovascular Medicine Unit, Department of Medicine, Imperial College, London, UK; 4 Queen Square Institute of Neurology, University College London, London, UK; 5 Children’s Heart Centre, Department of Pediatrics, BC Children’s Hospital–University of British Columbia, Vancouver, Canada; 6 School of Sport, Health and Exercise Science, University of Portsmouth, Portsmouth, England UK

**Keywords:** Artistic swimming, Autonomic conflict, Exercise, Syncope

Artistic (synchronized) swimming is an Olympic sport that combines skills of swimming, dance, weightlifting, cheerleading, and gymnastics. In competition, athletes are required to perform routines comprised of elaborate movements in the water, synchronized to music, which last from 2 to 5 min [1]. These require athletes to perform sustained vigorous exercise with intermittent prolonged breath-holds that can cumulatively account for 50% or more of their entire routine [2]. By combining breath-holding with near-maximal physical output, artistic swimming provides a significant and unique physiological stress. The specific nature of this stress is poorly understood, in part due to the challenge of making physiological measurements underwater, methodological inconsistencies across investigations conducted to date [3–7], and the rapid evolution of the sport’s complexity and difficulty since it was introduced into the Olympic program in 1984 [3]. The complex physiological paradigm of artistic swimming is further compounded by the simultaneous provocation of conflicting sympathetic “fight and flight” and parasympathetic “rest and digest” responses, with unpredictable effects on physiological responses (Fig. 1A). What is known is that, in rare instances, individuals performing artistic swimming routines have experienced episodes of loss of consciousness (syncope) in the water, raising the possibility of cardiac abnormalities, and posing an obvious safety risk. Here we describe what is known about the physiological demands and typical presentation of syncope during artistic swimming, including a recent case that occurred during the 2022 Fédération Internationale de Natation (FINA) World Championships (presently known as the World Aquatic Championships).

**Fig. 1:**
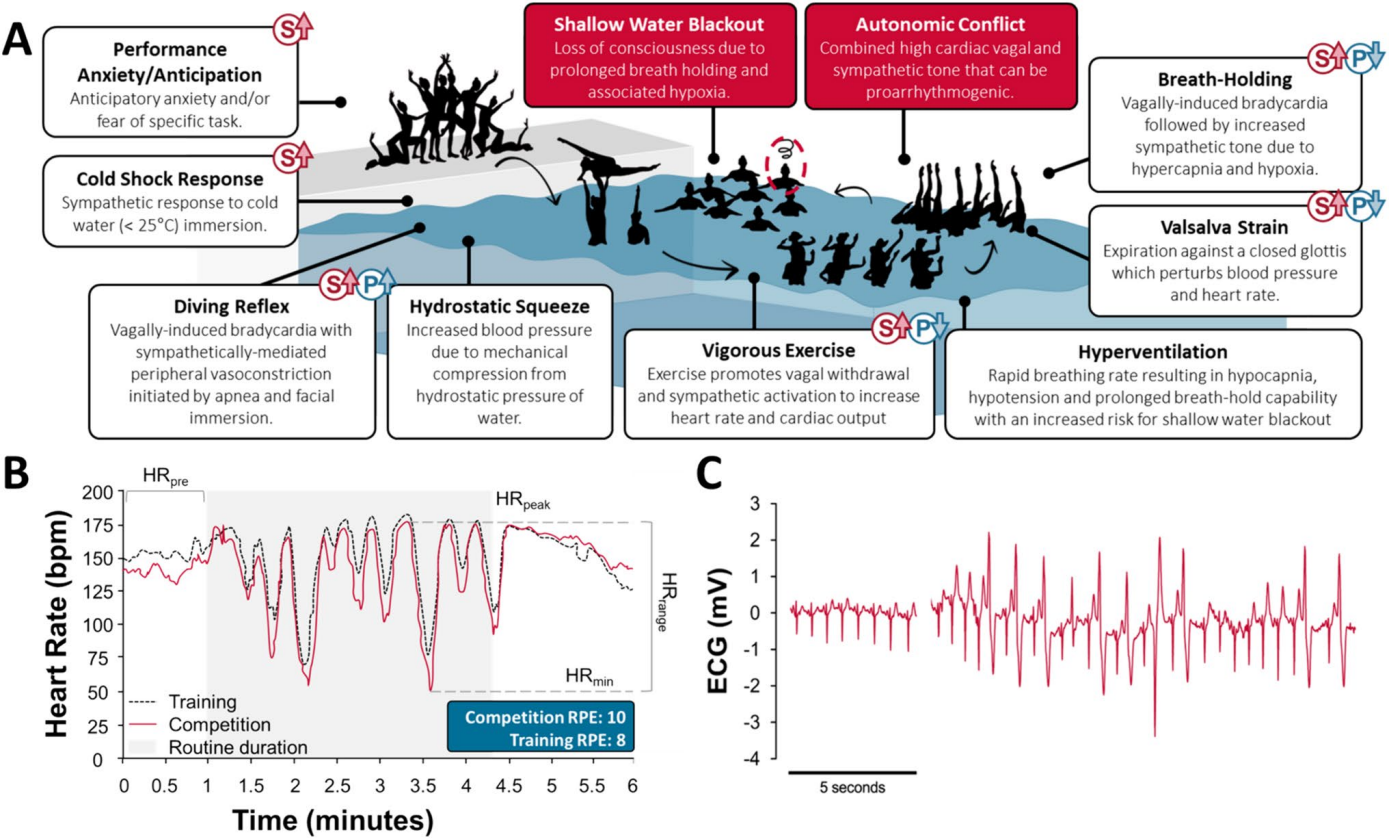
Physiological challenges associated with artistic swimming. **A** Artistic swimming provokes diverse physiological responses with complex and rapidly changing effects on cardiovascular regulation, with concurrent co-activation of sympathetic (S) and parasympathetic (P) pathways. **B** Example of heart rate responses during artistic swimming. Prior to starting the routine there is a substantive anticipatory heart rate increase (HR_pre_). During artistic swimming routines the heart rate fluctuates between near-maximal levels during exercise when breathing at the surface (HR_peak_) to sudden and profound decelerations (HR_min_) during excise when submerged and breath-holding. The heart rate range (HR_range_) is large and incongruous with the high level of physical activity undertaken, denoted by the high rating of perceived exertion (RPE; BORG-CR10 scale). Responses can be more profound during competition than in training. **C** Example electrocardiogram (ECG) during a routine training session initially showing exercise-induced sinus tachycardia, followed by cardiac arrhythmias while completing an underwater breath-hold activity, presumably associated with autonomic conflict. Multifocal ventricular ectopics can be seen, with some occurring in bigeminy. Figure 1B is adapted from Rodriguez-Zamora et al. [8]

## The physiological paradigm of artistic swimming

There is a paucity of data on the physiological responses to artistic swimming, with the limited available data focusing primarily on heart rate and blood lactate responses. Prior to performing or practicing a full routine there is an intense sympathetically-mediated anticipatory heart rate response (132 ± 15 bpm) [3] that reduces heart rate reserve, and can be exacerbated in cases of performance anxiety or during major competitions. During a routine, the heart rate quickly and progressively increases to near-maximal levels during periods of exercise at the surface while breathing (191 ± 15 bpm [3]); however with exercise during breath-holding there are abrupt decelerations in heart rate (82 ± 27 bpm [3]). In World Class athletes, minimum heart rates as low as 20 bpm have been observed during these periods [8], and cycles between maximal and minimal heart rate responses can occur within seconds (Fig. 1B). Concurrent with these fluctuating cardiac responses, peak lactate values (8.8 ± 1.4 mmol L^−1^) [3] indicate intense physical exertion; however these should be interpreted cautiously as peripheral vasoconstriction can compromise lactate removal [9], and there may be an effect of adaptation in reducing lactate responses with apneic training [10]. As confirmation of the high exercise intensity, ratings of perceived exertion during routines are high (7.9 ± 1.2; BORG-CR10 scale, corresponding to working “very, very hard” [8]). This high perceived exertion is heavily influenced by the number of immersions > 10 s during the routine, lactate levels, and minimum heart rate (but not maximum heart rate) during the routine [11], emphasizing that breath-hold frequency and duration contributes most to the perceived physiological difficulty of a given routine [11].

While the high heart rates observed likely reflect the exercise paradigm and associated sympathetic activation and parasympathetic withdrawal [12, 13], exacerbated by sympathetic activation due to the stress of competition [14] or fear of breath-holding [15], the low heart rate episodes predominantly reflect the mammalian diving response [3, 11, 16]. This response is triggered by facial immersion through cooling and wetting of sensory receptors predominantly located in the oronasal region [17]. The diving response is characterized by sympathetically-mediated peripheral vasoconstriction [17], with apnea and vagally-mediated bradycardia that counter the tachycardic heart rate response to exercise during periods of submerged breath-holding exercise. Diving responses are further enhanced by the initiation of breath-holding with submersion, which also provokes vagally-induced bradycardia [15, 17]. These responses are believed to promote the conservation of oxygen while underwater, and undergo sensitization with repeated exposure, producing profound cardiac slowing [18].

In most competitive pools, the cold shock response will oppose the diving response and contribute to tachycardia. This response occurs when cutaneous cold thermoreceptors are stimulated with sudden exposure to cold water (< 25 °C) immersion, and predominates for the first 1–3 min upon cold water immersion [19]. The cold shock response is characterized by sympathetically-mediated tachycardia and peripheral vasoconstriction, with an inspiratory gasp followed by hyperventilation [20]. While this response is most pronounced in water < 15 °C, competitive swimming pools are maintained at cold enough temperatures (approx. 22–27 °C) to activate, if relatively weakly, cold shock. Of note, repeated cold water exposure over time may reduce the cold shock response via predominantly central mechanisms [21], allowing for a more prominent diving response over time [22].

We evaluated routine components of medalists during the 2023 World Aquatics Artistic Swimming Championships and observed that between 59% and 81% of the routine is spent with the face submerged, necessitating substantial breath-holding. Accordingly, breath-holding is prioritized in artistic swimming training, with considerable emphasis on improving tolerance of the extreme discomfort of prolonged breath-holding through distraction [23], psychological skills training [23, 24], and training activities that enhance underwater breath-holding during physical activity independently of artistic swimming skills. Interestingly, much of the prior research on the physiology of breath-holding and breath-hold training has been conducted during low activity or resting states (e.g., free diving), while evaluation of the impact of exercise on responses or the role of psychological training during high-intensity physical exertion is little studied. The cumulative impact of repetitive near-maximal breath-holds with only a few seconds between to breathe at surface encountered in artistic swimming is not clear. The challenge with prolonged breath-holding, particularly during intense physical activity with high oxygen utilization, is that it poses a risk for shallow water blackout (loss of consciousness due to prolonged breath-holding and associated cerebral hypoxia) [25]. Indeed, alveolar oxygen tensions of 37 ± 8 mmHg have been reported immediately following completion of a free routine in national team athletes, associated with cyanosis and confusion, suggesting that potentially dangerous levels of hypoxia can occur during typical high-level routines [26]. Prior hyperventilation and associated hypocapnia in advance of breath-holding (either as a deliberate strategy or an involuntary response due to performance anxiety or oxygen debt [27]) promotes tachycardia and can prolong breath-holding capacity, but with the consequence of further reducing oxygen saturation while simultaneously decreasing cerebral blood flow and peripheral vascular resistance, posing an even greater risk of shallow water blackout [28, 29]. In addition, when breath-holding during exercise is accompanied by a Valsalva-like strain, for example when lifting team members above the water, profound blood pressure perturbations can occur that may provoke hypotension [29]. While this may be somewhat countered by hydrostatic squeeze and vasoconstriction from the cutaneous cooling and the diving response, unpredictable dynamic blood pressure responses during this profound exercise stress are likely and yet to date have not been studied.

The combination of these diverse physiological responses produces complex and rapidly changing effects on cardiovascular regulation (Fig. 1A), with co-activation of sympathetic and parasympathetic pathways. This warrants further investigation and evaluation as a physiological paradigm that is distinct from other exercise disciplines and from other aquatic sports in which prolonged breath-holding during intense exercise is not a dominant feature.

## Syncope during artistic swimming

Syncope (fainting) has been known to occur during artistic swimming routines in young, apparently healthy, high-performance athletes that are presumed free of risk factors for arrhythmia or sudden cardiac arrest. Community stakeholders shared that it is not uncommon for athletes training or coaching in preparation for national or international artistic swimming competitions to witness fainting, or perhaps experience it themselves. Typically, fainting occurs during, but near the end of the routine, when athletes are experiencing maximal fatigue, or during training drills designed to extend breath-hold time. However, syncopal episodes during slow, controlled, underwater elements known as “figures” are also reported. The actual prevalence or incidence of syncope in the sport has never been investigated and is not formally recorded. Some cases of fainting in artistic swimming athletes have been noted in the literature [27], with others reported in the media [30, 31], likely due to the high-profile nature of international competitions. One case at the 2022 FINA World Cup was particularly well described in the media and featured typical characteristics of these episodes, occurring at the end of the free solo routine, following extreme exertion with prolonged breath-holding, and when the athlete was maximally fatigued. The athlete was rescued unconscious from the bottom of the swimming pool with an obvious drowning risk. Of note, the same athlete had experienced syncope during both training and competition previously in similar circumstances [30]. There are many other similar examples in the field [27]. As in many of these episodes, extensive medical and sport-specific evaluations have failed to identify any overt cardiovascular or neurological abnormalities [32], suggesting it may reflect specific features of the sport, rather than the athlete.

Although the specific causes of fainting during artistic swimming are unknown, there are two key physiological mechanisms that might be at play. Shallow water blackout secondary to prolonged breath-holding, submersion, and associated hypoxia (particularly during ascent following inversion) may contribute to fainting susceptibility, as it does in other breath-holding sports such as free diving. Certainly, artistic swimmers hold their breath intermittently throughout their routine; each breath-hold is typically 10–15 s but sometimes extends beyond 25–30 s for high-performance athletes. Critically, these breath-hold phases are accompanied by vigorous exercise and repeated throughout the routine with limited time (a few seconds) at the surface to breathe before the next breath-holding phase, such that the impacts of breath-holding accumulate and account for a substantial portion of each routine. For example, in a sample of routines ranking 1–5 performed at Olympic or World Aquatics events between 2020 and 2023, underwater choreography cumulatively accounted for 60 ± 1%, 63 ± 1%, and 69 ± 2% of the routine duration for senior technical teams, duets, and solos, respectively.

One emerging and under-appreciated contributor to syncope during artistic swimming routines is a phenomenon known as “autonomic conflict,” whereby simultaneous cardiac sympathetic and parasympathetic activation with conflicting influences on the heart can trigger cardiac arrhythmias [22]. Indeed, we have observed evidence of cardiac arrhythmias during underwater breath-hold training, even during a relatively mild training session, with induction of frequent multifocal ventricular ectopics, including episodes of bigeminy, in an otherwise healthy artistic swimming athlete (Fig. 1C). Syncope associated with exercise is well documented, but because of the unique autonomic stimulus in aquatic sports, the cause is more likely to be arrhythmic in these cases. Indeed, others have shown that, in general, autonomic conflict-induced cardiac arrhythmias during cold water immersion are common and underappreciated [33, 34]. During underwater choreography, parasympathetic drive from breath-holding and the diving response increases concurrently with high sympathetic tone elicited primarily from exercise responses, performance anxiety, and cold shock [14, 15]. This phenomenon is more likely with prolonged breath-holding and within 10 s of the release of a breath-hold [34, 35], and can provoke cardiac arrhythmia and a subsequent loss of consciousness [36]. This could be particularly problematic in athletes with underlying and possibly undiagnosed cardiac conditions that might be more susceptible to autonomically conflicting stimuli [22]. Of note, community stakeholders shared that they are not aware of established cardiac screening practices for high-performance athletes, but this may be a beneficial practice in athletes that have experienced syncope during training or competition.

Despite the occurrence of syncope in artistic swimming and the uncertain cause of these episodes, breath-holding during immersed exercise is a key risk factor for the two most likely drivers of these events. This is notable because underwater breath-holding is embedded into the sport, and is further prioritized following recent changes in scoring in which difficulty scores are primarily allocated during underwater choreography. The concern with this change is that longer durations under water would receive higher scores, with an obvious impact of encouraging longer breath-hold durations, pushing the boundaries of safe participation [26] and highlighting the challenges with safely training this key skill for the sport. We are encouraged to see that these challenges are being recognized by the community, with the instigation of apnea monitoring at the junior and lower levels and the initiation of maximal apnea durations and bonus scoring for routines below the apnea maximum in these categories. However, even with these new breath-holding considerations, the total time breath-holding during a routine remains long, and whether these safety precautions will also be applied to senior athletes is, at present, unclear.

Other factors that might further predispose to syncope during artistic swimming relate to concurrent stress responses. Heart rate ranges can be larger during competition than training (Fig. 1B) [8]. This likely reflects athletes pushing boundaries when striving for excellence in competition, accompanied by some measure of performance anxiety, and suggests that syncopal events may be more common during competition than training [37]. Certainly, acute anxiety increases the magnitude of both cardiac and respiratory components of the cold shock response [38, 39], leading to more pronounced increases in heart rate with cold water exposure, even following habituation [38]. Further, repeated anxiety impairs habituation of cold shock, possibly supporting a link between anxiety and an increased risk of autonomic conflict during artistic swimming [40]. This is important because in addition to the understandably stressful nature of the competition environment, our community stakeholders have emphasized that athletes often experience anxiety around breath-holding, even during training sessions.

An additional complicating factor might relate to the presence of relative energy deficiency in sport, which has been noted in artistic swimmers [41], alongside a high prevalence of disordered eating (18–45%) [42], both of which are associated with increased risk for syncope [43, 44]. This is important as relative energy deficiency and/or disordered eating may be more prevalent in artistic swimming because it is an sport judged on aesthetic, with an emphasis on a lean and uniform physique among team members [45].

Finally, some community stakeholders noted that there is stigma associated with fainting that discourages reporting, making it hard to identify, address, and manage syncope risk. Education around fainting risks alongside practical guidelines to manage and respond to fainting events were noted as a priority.

## Conclusions

Artistic swimming is a physically demanding sport with a complex physiological paradigm. In rare cases, artistic swimming is associated with syncopal events, typically in young, apparently healthy, high-performance athletes, and these pose an obvious safety risk. The mechanisms underlying these events deserve further study, but likely relate to shallow-water blackout and/or cardiac arrhythmias associated with autonomic conflict, both of which are exacerbated by longer breath-hold durations. Evidence-based practical recommendations on how to respond to a fainting event and the management of recurrent fainting in artistic swimming athletes are needed.
